# Post-traumatic stress among adolescents following the October 7th attack in Israel: implications for mental health policy and planning

**DOI:** 10.1186/s13584-025-00740-1

**Published:** 2025-12-12

**Authors:** Tehila Refaeli, Agat Sold, Limor Aharonson-Daniel

**Affiliations:** 1 The Charlotte B. and Jack J. Spitzer Department of Social Work, Ben-Gurion University of the Negev, Beer-Sheva, 8410501 Israel; 2 School of Public Health, Faculty of Health Sciences, Ben-Gurion University of the Negev, Beer-Sheva, 8410501 Israel; 3 PREPARED Center for Emergency Response Research, Ben-Gurion University of the Negev, Beer-Sheva, 8410501 Israel

**Keywords:** Adolescents, War exposure, Media exposure, Indirect trauma exposure, Resilience, Mental health policy, Resilience planning

## Abstract

**Background:**

The October 7th, 2023, attack in Israel and the ensuing war exposed Israeli youth to intense and prolonged traumatic stress. This study aimed to: (1) examine the prevalence of probable post-traumatic stress disorder (PTSD) among Israeli adolescents, and (2) identify associated risk and resilience factors for PTSD symptoms within the context of prolonged exposure to threat and uncertainty due to ongoing war and conflict.

**Methods:**

A cross-sectional digital survey was conducted between October and December 2024. Data were collected from 744 adolescents aged 14–18 years, including the Child PTSD Symptom Scale (CPSS-SR-5), questions about direct war-related consequences and indirect exposure. Personal resilience was assessed using the Connor-Davidson Resilience Scale. Social support was measured using the Multidimensional Scale of Perceived Social Support (MSPSS). Background questions addressed age, gender, health, and adverse events in the past year. Hierarchical multiple regression analysis was used to examine the contribution of background variables, war-related effects, indirect exposure, and resilience factors to PTSD symptoms.

**Results:**

The study revealed that 41.9% of participants met the probable diagnostic threshold for PTSD. Gender (female), poorer health status, and previous adverse experiences correlated with increased symptom severity. Among the direct effects, economic status deterioration and direct war impact emerged as significant predictors. Among the indirect exposure factors, frequent news consumption was associated with higher PTSD symptoms. Personal resilience and family support were associated with reduced PTSD symptoms, although resilience factors did not significantly moderate the adverse impacts of war-related experiences.

**Conclusions:**

The high prevalence of probable PTSD among Israeli adolescents in the wake of the October 7th, 2023 attack suggests an urgent need for action. Evidence-informed mental health policies and services should prioritize adolescents, with targeted support for high-risk groups. Our findings stress the need for a national mental health policy to incorporate youth-centered trauma response systems, including interventions for indirect media trauma. Strengthening personal resilience and bolstering family support systems is vital in order to mitigate the lasting psychological impact of war-related trauma on youth.

**Supplementary Information:**

The online version contains supplementary material available at 10.1186/s13584-025-00740-1.

## Introduction

On October 7th 2023, Hamas launched a brutal attack on Israel. This attack resulted in over 1,200 deaths, thousands of injuries, and the abduction of more than 240 hostages [1]. An ongoing war has since ensued. The consequences for children and adolescents have been particularly devastating [2, 3]. In the attack, 53 children were killed and 36 were abducted to Gaza. In the aftermath, 870 lost one parent, and 23 lost both [2]. Many of the victims were young people at a nature festival, contributing to the fact that 1,014 children lost at least one sibling.

Beyond the immediate casualties, more than 19,900 children have been recognized as terror victims, primarily due to emotional trauma, and 1,873 have developed physical or mental disabilities as a direct result of the attacks and ongoing war. Furthermore, approximately 300,000 children have parents serving in reserve duty [2]. In terms of evacuation, 53,524 children were evacuated from border communities [4] experiencing displacement, separation from social networks, and adaptation challenges in new environments [3]. These evacuated children faced stressors that extended beyond direct exposure to terror attacks.

The acute exposure to such extreme adversity has had profound mental health consequences, with emerging evidence documenting increased post-traumatic stress disorder (PTSD) among adults [5, 6]. PTSD is characterized by intrusive memories, hyperarousal, avoidance of trauma-related stimuli, and persistent psychological distress, which significantly impair individual functioning and quality of life [7].

In the current study, we focused on adolescents in Israel, exploring the rates of probable PTSD and factors associated with symptoms one year after the October 7th attack and during the continuing war. To our knowledge, this study represents the first investigation of these issues among Israeli adolescents after October 7th. Examining adolescents’ PTSD symptoms one year after the October 7th attack is important, as chronic posttraumatic reactions often become evident months after exposure [7]. An extended review of studies on posttraumatic stress symptom trajectories among children and adolescents demonstrated that although many recover, a substantial subgroup has chronic or even delayed trajectories [8]. Evidence from war-affected cohorts likewise indicates heterogeneous courses. In Croatia, post-traumatic stress among war-exposed youth declined over 30 months, yet persisted among the highly exposed [9]. Additionally, among Israeli adolescents living under ongoing missile attacks, high baseline probable-PTSD rates were followed by symptom reductions over time within a school-based follow-up [10]. These studies underscore the need for long-term assessments of PTSD symptoms and risk and resilience factors. The current study can thus provide insights for developing targeted interventions in high-income countries exposed to terror and armed conflict.

### Risk and resilience factors for PTSD

Multiple risk factors have been associated with elevated PTSD among children and adolescents. Gender has emerged as a significant predictor, with female adolescents reporting higher symptom severity than their male counterparts [11]. Additionally, among adolescents, older age has been identified as a key risk factor for PTSD across different types of traumas, including personal and natural disasters [12].

Among adults, pre-attack factors associated with the risk for PTSD include economic hardship and compromised health status [5, 13]. Longitudinal studies among adults have demonstrated bidirectional effects in the health-PTSD association, whereby poor physical health predicts subsequent PTSD symptom severity, and conversely, PTSD exacerbates physical health deterioration [14–16]. In addition, prior exposure to traumatic events has been recognized as a PTSD risk factor for both adults and adolescents [17]. Proximity to the traumatic event further increases PTSD symptom likelihood, with direct exposure posing a substantial risk [13, 18]. Although these findings are well-established in adult populations (ages 18–85), research examining whether these factors similarly predict PTSD among adolescents exposed to sustained conflict remains limited.

Media exposure has emerged as another critical risk factor for PTSD. Research demonstrates that indirect exposure to traumatic events through media coverage can elicit acute stress responses and PTSD symptoms in adolescents [19, 20] (e.g., after 9/11) [21]. Following the October 7th attack, a survey of the Israeli National Headquarters for the Protection of Children Online [4] indicated that nearly two-thirds of adolescents encountered highly distressing media content. Notably, those with greater media exposure were twice as likely to experience psychological distress and engage in maladaptive coping behaviors, including alcohol consumption [2]; however, the effect on PTSD was not explored.

Beyond risk factors, the literature emphasizes resilience factors as protective elements against PTSD. Personal resilience refers to a set of skills, values, and characteristics that enable individuals to withstand challenges, stress, and trauma, fostering their ability to adapt to new circumstances [22]. Subsequently, research has demonstrated an association between personal resilience and lower PTSD levels among adolescents experiencing diverse stressful and traumatic events [23, 24].

Social support has emerged as another pivotal resilience factor in reducing PTSD responses following terrorism and war exposure among adolescents [25]. A comprehensive meta-analysis revealed that higher levels of social support from family and friends correlated with lower PTSD symptoms among children and adolescents exposed to personal and collective traumas. This protective effect was most pronounced for children confronting severe, prolonged trauma, underscoring the significant role of social support in psychological adaptation [26].

## The current study

This study aimed to (1) examine the prevalence of probable PTSD among Israeli adolescents and (2) identify risk and resilience factors associated with PTSD symptoms among Israeli adolescents exposed to the October 7th, 2023 events and their aftermath. Whereas previous studies have predominantly examined PTSD predictors in the immediate aftermath or a short time after traumatic events [27, 28], our research focused on PTSD symptoms one-year post-terror attack and during ongoing exposure to continuous threat. This timeframe is critical for distinguishing chronic PTSD from acute stress reactions. Whereas acute PTSD symptoms emerge within the first three months post-trauma, chronic PTSD persists beyond this period and represents more severe, treatment-resistant distress [7]. We examined one-year post-attack PTSD in adolescents under continuing threat—an interval at which chronic and delayed trajectories begin to differentiate [9, 10]. Our aim was to provide both theoretical and practical insights into how resilience factors protect mental health under sustained exposure conditions, as the study was conducted while the conflict was ongoing.

More specifically, we tested the following hypotheses:

1. Participants who experienced direct war-related effects would report higher levels of PTSD symptoms than those who did not.
2. Participants who experienced indirect exposure would report higher levels of PTSD symptoms than those who did not.
3. Higher levels of personal resilience and social support would be associated with lower levels of PTSD symptoms.
4. Personal resilience and social support would moderate the associations between direct war effects and PTSD symptoms, and between indirect exposure and PTSD symptoms.

## Method

### Participants and procedure

Data were collected between October and December 2024, one-year post-October 7th and during the ongoing ensuing war. The sample was obtained through non-probability sampling and recruited online. A digital quantitative survey was distributed via WhatsApp groups and promoted through targeted advertisements on Instagram and Facebook. The study relied on a convenience sample, which is not representative of the broader population. The survey took approximately 15 min to complete. Participants younger than 18 provided parental consent via an electronic signature before gaining access to the survey. To encourage participation, monetary prizes were raffled among respondents. The final sample included 744 adolescents. Participants were between 14 and 18 years old, inclusive (M = 17.23, SD = 1.17).

### Measurements

**Post-traumatic stress disorder (PTSD)** was assessed using the Child PTSD Symptom Scale-Self-Report for DSM-5 (CPSS-SR-5; [29]), a 20-item measure based on DSM-5 diagnostic criteria. Participants were asked to refer to symptoms following the October 7th, 2023 events and the war, and to rate symptom frequency over the past month on a 0–4 scale (“not at all” to “almost always”), yielding total scores from 0 to 80, with higher scores indicating greater severity. Following standard guidelines, scores ≥ 31 indicated probable PTSD. Although not diagnostic, the CPSS-SR-5 is widely used in research and showed high reliability in this study (α = 0.93).

**War-related effects** included three questions: (1) family’s economic status following the war (1 = deteriorated, 0 = improved or unchanged); (2) family members’ enlistment to reserve service (1 = yes); and (3) direct negative war impact, assessed by determining whether participants experienced at least one adverse event from five categorical options (0 = no, 1 = yes). Participants were asked, “Were you directly affected by the war?” with response options: (1) “Myself/my family,” (2) “My home,” (3) “My community,” (4) “My friends,” and (5) “Other (please specify).” Participants endorsing at least one experience were coded as having a direct war impact (1 = yes) versus no direct impact (0 = no). Among those selecting “Other,” responses predominantly included physical and mental injuries sustained by relatives or individuals close to the participants or their families, as well as knowing someone close to them who was killed or kidnapped. To explore moderation effects, we created an index variable summing all war-related effects (range 0 to 5).

**Indirect exposure** included two questions: (1) Around October 7th and afterwards, did you encounter graphic content that was difficult to watch on digital media? (2) During the war, did you feel emotionally overwhelmed or flooded by consuming news and information? (1 = yes). To explore moderation effects, we created an index variable equal to 1 if media exposure or overwhelming news consumption was reported.

**Personal resilience** was measured using the 10-item Connor-Davidson Resilience Scale [22], with responses ranging from 0 to 4 (“not true at all” to “true nearly all the time”). The total score was the sum of the 10 items (α = 0.86).

**Social support** was measured using the Multidimensional Scale of Perceived Social Support (MSPSS) [30]. We used the family and friends sub-scales and a general support scale that included both subscales and four additional items (12 items in total). Responses were rated on a 7-point scale (“not suitable at all” to “very suitable”), with scores calculated as the average of items (α = 0.93 for friends, α = 0.87 for family, α = 0.88 overall).

**Background questions** addressed participants’ age, gender, health status (1 = very bad to 5 = very good), and experiencing other adverse event/s not related to the war during the previous year (e.g., injury, accident, or sickness of a close one) (1 = yes).

### Data analysis

Data were analyzed using SPSS version 29. Bivariate analyses (t-tests and Pearson correlations) examined associations between study variables. A hierarchical multiple regression was then performed to identify PTSD predictors. The regression comprised four steps. Step 1 encompassed background variables (gender, age, health status, and another stressful event). Steps 2 and 3 included PTSD risk factors. Specifically, Step 2 included variables related to the war’s direct consequences (economic status, family member drafted, and harm following the war), and Step 3 incorporated indirect exposure (exposure to graphic content in the media and overwhelming consumption of news). Step 4 addressed resilience factors, including personal resilience, family support, and friend support. Finally, using PROCESS v3.1 macro for SPSS [31] we examined the moderating effects of the resilience resources (personal resilience, family and friend support, and general support) on the associations between the war-related effects, indirect exposure indexes and PTSD. The extent and pattern of missing data were examined. Little’s Missing Completely at Random (MCAR) test was non-significant, χ²(3) = 2.22, *p* >.05, supporting the assumption that missingness was completely at random. As the rate of missing values was minimal, we proceeded with the analyses without applying imputation techniques.

### Ethics

Ethical approval was obtained from the ethics committee of the authors’ university. Participants provided electronic consent to participate in the study. For minors under 18, one parent also provided consent. We avoided asking directly about the death or kidnapping of close ones due to the use of an online questionnaire and the inability to provide immediate emotional support during or following participation. We therefore articulated questions using the term “affected,” which enabled participants to refer to their subjective experiences without exposure to potentially triggering language. In cases of emotional distress following completion of the questionnaire, we provided the authors’ contact information (phone numbers and email addresses) as well as phone numbers for youth support hotlines.

## Results

Based on the established cutoff score of 31 on the CPSS-SR-5 [29], 41.9% of the participants met the threshold for probable PTSD, indicating a substantial probable prevalence of trauma-related distress within the sample.

An analysis of the associations between PTSD total score and the background and risk variables is provided in Table 1. Post-traumatic symptoms were significantly higher among girls (M = 34.44, SD = 16.65) than boys (M = 20.89, SD = 15.98), t = 13.07, *p* <.001. Age was not significantly associated with PTSD symptoms (*r* =.04, n.s.). Better health status correlated with lower PTSD symptoms (*r* = −.33, *p* <.001). Youth who experienced other adverse event/s had significantly higher PTSD scores (M = 35.05, SD = 16.88) than those who did not (M = 27.70, SD = 17.35), t = 6.50, *p* <.001.

Respondents reporting worsened economic status due to the war exhibited higher PTSD scores (M = 36.45, SD = 18.67) than those whose status remained unchanged or improved (M = 27.38, SD = 16.27), t = 5.93, *p* <.001. No significant association was found with having a family member in a reserve service. Those experiencing a direct negative war impact reported higher PTSD symptoms (M = 35.46, SD = 18.35) than those without such an impact (M = 26.46, SD = 16.22), t = 6.22, *p* <.001. In addition, participants who had more war-related effects reported more PTSD symptoms, r(744) = 0.27, *p* <.001.

Exposure to harsh media content was associated with higher post-traumatic symptoms (M = 30.09, SD = 17.78 vs. M = 27.60, SD = 15.75), t = 1.94, *p* <.05. Additionally, respondents reporting overwhelming news consumption demonstrated significantly higher PTSD scores (M = 35.37, SD = 15.52) than those who did not (M = 23.65, SD = 16.56), t = 9.81, *p* <.001. Finally, 81.2% of the respondents reported either exposure to harsh media content or overwhelming news consumption, and the exposed group reported significantly higher PTSD symptoms (M = 30.74, SD = 17.16) than those who were not exposed to media or overwhelming news (M = 23.04, SD = 15.83), t = 4.78, *p* <.001.

**Table 1:** Frequencies and PTSD mean scores by background variables and risk factors

Variable	Category	*n* (%)	M	SD	t/*r*
Gender	Male	255 (34.3%)	20.89	15.98	13.07***
	Female	489 (65.7%)	34.44	16.65	
Age					0.04
Health status (1-very bad- 5-very good)					− 0.33***
Another adverse event/s	Yes	215 (29.3%)	35.05	16.88	6.50***
	No	519 (70.7%)	27.70	17.35	
Economic status	Deteriorated	152 (20.6%)	36.45	18.67	5.93***
post-war	Other	587 (79.4%)	27.38	16.27	
Family member in reserves	Yes	219 (29.5%)	29.58	16.62	0.37
	No	524 (70.5%)	29.07	17.37	
Direct war impact	Yes	187 (27.6%)	35.46	18.35	6.22***
	No	490 (72.4%)	26.46	16.22	
Sum of war-related effects					0.27***
Media exposure	Yes	489 (66.3%)	30.09	17.78	1.94*
	No	249 (33.7%)	27.60	15.75	
Overwhelming news consumption	Yes	349 (51.7%)	35.37	15.52	9.81***
	No	373 (48.3%)	23.65	16.56	
Indirect exposure	Yes	598 (81.6)	30.74	17.16	4.78***
	No	135 (18.4)	23.04	15.83	

*p <.01, ***p <.001

Table 2 presents the Pearson correlation coefficients between personal resilience, social support variables, and PTSD symptoms. PTSD symptoms were negatively correlated with resilience resources, indicating that higher levels of personal resilience and social support were associated with lower PTSD symptom severity. Specifically, personal resilience demonstrated the strongest negative correlation with PTSD (*r* = −.42, *p* <.001). Family support (*r* = −.31, *p* <.001), friend support (*r* = −.25, *p* <.001), and overall social support (*r* = −.33, *p* <.001) were also negatively associated with PTSD.

**Table 2:** Correlations between resilience factors and PTSD (*N* = 734–744)

Variable	1	2	3	4	5
1 **Personal Resilience**	-				
2 **Family Support**	0.30***	-			
3 **Friend Support**	0.39***	0.40***	-		
4 **Social Support**	0.41***	0.81***	0.86***	-	
5 **PTSD**	− 0.42***	− 0.31***	− 0.25***	− 0.33***	-

***p <.001

### Multivariate regression predicting PTSD symptoms

A hierarchical regression analysis examined the contribution of background variables, direct war-related consequences, indirect exposure, and resilience resources to assess PTSD symptoms. The final model explained 39.3% the variance in PTSD symptoms, F(12, 662) = 36.72, *p* <.001 (Table 3).

In Step 1, background variables accounted for 21.8% of the variance. Gender emerged as a significant predictor, with girls reporting higher levels of PTSD symptoms than boys (β = −0.30, *p* <.001). Additionally, poorer health status was associated with higher symptom severity (β = −0.25, *p* <.01). Exposure to other adverse events in the past year also significantly predicted greater PTSD symptoms, whereas age was not a significant predictor (β = 0.13, *p* <.001).

In Step 2, the direct consequences of the war contributed an additional 5.8% to the explained variance. Adolescents whose families’ economic status deteriorated due to the war reported significantly higher PTSD symptoms than those who did not experience negative changes (β = 0.14, *p* <.001). Participants who experienced at least one direct negative impact of the war also exhibited greater symptom severity (β = 0.19, *p* <.001). Having a family member on reserve duty was not a significant predictor of PTSD levels (β = 0.01, n.s.).

In Step 3, indirect exposure factors contributed an additional 2.6% to the explained variance. Overwhelming news consumption remained significant after controlling for other factors, with those experiencing excessive news exposure reporting greater distress (β = 0.19, *p* <.001). In contrast, graphic media exposure was not a significant predictor when controlling for other factors (β = 0.05, n.s.).

In Step 4, resilience resources contributed an additional 8.3% to the explained variance. Higher personal resilience (β = − 0.24, *p* <.001) and greater family support (β = − 0.13, *p* <.001) were linked to lower PTSD symptom severity. However, friend support did not emerge as a significant predictor (β = − 0.04, n.s.). Across all steps, significant predictors from earlier models remained robust, highlighting the central role of background variables, followed by resilience resources, in predicting PTSD symptoms.

Lastly, we examined the moderation effects of the three resilience resources on the associations between the war-related effects, indirect exposure indexes and PTSD using PROCESS macro [31]. No moderation effects were statistically significant (see Appendix A), indicating that resilience factors did not mitigate the negative association of the risk factors with PTSD.

**Table 3:** Multivariate hierarchical regression analysis of the contribution of the study variables to predicting PTSD symptoms

	*Model 1*			*Model 2*			*Model 3*			*Model 4*			
	B	S.E.B	B	B	S.E.B	B	B	S.E.B	B	B	S.E.B	B	
**Background variables**													
Gender (Female)	10.96	1.28	0.30***	10.93	1.24	0.30***	9.11	1.26	0.25***	7.28	1.21	0.20***	
Age	0.70	0.50	0.04	0.70	0.49	0.05	0.42	0.48	0.03	0.63	0.45	0.04	
Health	−5.62	0.81	− 0.25***	−5.00	0.79	− 0.22***	−4.54	0.77	− 0.20***	−2.07	0.77	− 0.09**	
Another adverse event/s (Yes)	4.72	1.32	0.13***	4.07	1.27	0.11***	3.72	1.24	0.10***	3.16	1.18	0.08***	
**Consequences of the War**													
Economic status following the war (Less good)				6.03	1.43	0.14***	5.67	1.4	0.13***	4.17	1.33	0.10***	
Family member drafted (Yes)				0.45	1.26	0.01	0.06	1.23	0.02	0.92	1.16	0.02	
Direct war impact (Yes)				7.18	1.29	0.19***	6.65	1.27	0.17***	7.1	1.19	0.19***	
**Indirect Exposure**													
Media Exposure (Yes)							1.71	1.19	0.05	1.72	1.11	0.05	
Overwhelming News Consumption (Yes)							6.63	1.19	0.19***	6.15	1.12	0.18***	
**Resilience Resources**													
Personal Resilience										−0.56	0.08	− 0.24***	
Family support										−1.63	0.43	− 0.13***	
Friend support										−0.48	0.39	− 0.04	
*Adjusted R²*	0.22			0.28			0.31				0.39		
*R² Change*	0.22***			0.06***			0.04***				0.08***		

**p <.01, ***p <.001; Bold=statistically significant

## Discussion

The October 7th attack and subsequent prolonged war have placed a significant psychological burden and taken a great toll on the Israeli population [5, 6], with particularly devastating consequences for children and adolescents [2]. Israeli adolescents have experienced extensive exposure to traumatic events, including direct and indirect exposure to murder and death, injuries to close relatives, family members’ military service, missile attacks, and persistent security threats [2, 3]. As far as we know, to date, no comprehensive studies have systematically explored the mental health landscape of Israeli adolescents under such sustained and acute threat conditions. Moreover, the complex interplay of risk and protective factors associated with mental health—specifically PTSD—within this context of severe, prolonged adversity remains underexamined.

Our findings reveal an alarming prevalence of probable PTSD based on a screening tool, with over 40% of adolescents meeting the diagnostic threshold—a rate substantially higher than established benchmarks in trauma research. This exceptionally high prevalence represents the central and most striking finding of the study. For comparative context, a comprehensive meta-analysis of trauma-exposed children and adolescents previously documented an overall PTSD rate of 15.9%, using clinical assessments for PTSD [32]. Similarly, studies examining adolescents’ psychological responses to events such as the 9/11 attacks and the COVID-19 pandemic reported PTSD symptomatology rates of around 10% using screening tools [33, 34]. Still, studies from the Syrian conflict and regarding Palestinian children and adolescents indicated similar or higher rates of PTSD, using both clinical assessments and screening tools [35, 36]. The markedly elevated rates in these studies and our study underscore the profound and potentially long-lasting mental health implications of sustained, severe traumatic exposure. These findings not only illuminate adolescents’ psychological vulnerability during prolonged conflict but also emphasize the urgent necessity of identifying nuanced risk and protective factors, as discussed below, to mitigate PTSD symptomatology.

Longitudinal research with war-exposed adolescents demonstrates heterogeneous PTSD courses rather than uniform decline [10, 32]. In Croatia, symptoms generally lessened across 30 months but remained elevated among youth with a greater trauma “dose” and ongoing stressors [9]. In Israel, adolescents living under chronic rocket fire showed a high baseline prevalence of probable PTSD with subsequent reductions across school-based follow-ups [10]. Together, these data suggest that our one-year assessment likely included adolescents on recovery trajectories and a non-trivial subgroup on chronic or delayed-onset paths, especially given continued exposure, repeated reminders, and resource loss following October 7th, 2023.

Prior research has identified gender and health status as PTSD risk factors [17]. Our findings align with this pattern: background variables, particularly female gender and poor physical health, emerged as the strongest predictors of PTSD symptoms. Still, the substantial contribution of pre-trauma factors, accounting for 22.2% of the variance, diverges from earlier findings. The Claxton et al. meta-analysis [37] examined pre-, during, and post-traumatic event risk factors for depression among trauma-exposed children and adolescents. The Claxton et al. meta-analysis [37] revealed only small effect sizes for pre-trauma variables. This discrepancy may reflect distinct etiological pathways underlying depression versus PTSD, or alternatively, the unique circumstances of Israel’s severe terror attacks followed by sustained warfare. Under conditions of chronic instability and ongoing threat, individual vulnerabilities and protective factors unrelated to specific traumatic events may exert a disproportionate influence on psychological outcomes.

Regarding physical health specifically, as mentioned, reciprocal relationships between PTSD and physical health have been documented in previous longitudinal studies [14–16]. The present findings among adolescents underscore the importance of further examining physical health as a pre-trauma vulnerability factor, not solely as a consequence of trauma exposure, particularly in developmental populations where such mechanisms may differ from those observed in adults.

Although previous studies have found age to be a significant predictor of PTSD [38], we did not observe this association in our sample. This null finding may be attributable to the restricted age range (14–18 years) and the uneven age distribution within our sample, with younger adolescents being underrepresented. This limited variability may have constrained our ability to detect age-related effects.

In the current study, we explored both direct and indirect war effects, some of which have been less extensively investigated in relation to PTSD. The substantial contribution of deteriorated economic status to PTSD symptoms underscores the need for a more comprehensive understanding of war’s economic impact on adolescents’ mental health. Approximately a quarter of participants reported negative changes in their family’s economic situation, potentially resulting from parental job losses due to evacuation, reserve duty, or property damage. According to conservation of resources (COR) theory, deterioration in economic status constitutes a major loss of resources, which is a primary source of stress [39]. Such loss may trigger a spiral of further losses [40], undermining stability and control, and thereby intensifying adolescents’ traumatic experience. Building on previous research demonstrating strong associations between economic status and adolescent mental health [41], we posit that adolescents are acutely aware of these economic shifts. Such awareness may add to the traumatic experience, potentially generating additional anxiety about the potential inability to meet basic needs.

Our findings regarding the role of direct war harm as a factor associated with elevated PTSD align with previous research [13, 18]. The absence of an association between family member reserve duty and adolescent PTSD symptoms may reflect the Israeli sociocultural context, where military service and reserve duty are normalized civic experiences. This normalization may have attenuated adolescents’ threat perceptions and served as a protective factor. However, our measure did not differentiate between combat and non-combat reserve roles, a distinction that may have been critical, as family member involvement in active combat operations could have differential effects on adolescent posttraumatic symptoms compared to combat support functions.

Additionally, although some studies have highlighted the negative effects of indirect exposure generally [19, 20], and specifically in the Israeli context [2], our findings offer nuanced insights. Following the October 7th attack and subsequent war, social media and news were saturated with graphic images, making exposure nearly unavoidable. Consequently, in the current study, most participants reported encountering graphic content and experiencing overwhelming news consumption. Viewed alongside longitudinal war cohorts [9, 10], sustained media-related overwhelm under ongoing threat may put adolescents at heightened risk for non-resolving or delayed-onset PTSD when reminders persist. Interestingly, although our bivariate analysis showed associations between both graphic content exposure and overwhelming news consumption with PTSD, the regression analysis revealed only overwhelming news consumption as a significant predictor. This finding may suggest that the psychological outcomes stem not merely from exposure but from participants’ emotional responses to such content. Such a perspective provides critical insights for potential interventions in scenarios where avoiding graphic media is impossible. Although the ideal approach would be reducing exposure to graphic and terrifying news, adult-mediated media consumption could potentially mitigate its negative psychological impacts.

The Claxton et al. [37] meta-analysis highlighted post-trauma variables, particularly social support, as having a large effect size in assessing depression after traumatic events. In contrast, our findings reveal less encouraging findings regarding personal resilience and family support in mitigating PTSD symptoms. Although resilience factors were the second most significant contributor to PTSD, the results were nuanced. Friend support did not significantly reduce PTSD symptoms, and personal resilience emerged as more important than family support. These findings suggest that during prolonged, severe trauma, social support networks—consisting of individuals sharing the same adverse reality—may be compromised. The ongoing war potentially damages protective factors that typically buffer stress in routine or short-term challenging circumstances [42]. Moreover, the inability of resilience factors to mitigate war-related negative influences associated with PTSD in the current study can also be explained by the fact that sustained traumatic exposure may overwhelm traditional psychological and social protective mechanisms.

### Limitations and future directions

Although our findings contribute to understanding risk and resilience factors for PTSD among adolescents exposed to prolonged traumatic events, several limitations must be acknowledged. The cross-sectional study design inherently limits our ability to establish causal relationships between the identified factors and PTSD symptoms. This limitation is especially relevant to the physical health-PTSD association. Moreover, the questionnaire’s online distribution created a sampling bias, underrepresenting groups with limited digital access, such as the ultra-Orthodox community and socioeconomically disadvantaged communities. Additionally, the digital recruitment strategy may have attracted participants with higher media consumption patterns, potentially inflating media-related findings. Given their relatively small proportion in the population, our sample included an insufficient number of evacuated adolescents; therefore, this variable was excluded, highlighting the need for future research. A further limitation is that the study did not assess participants’ trauma history, such as adverse childhood experiences, despite evidence that prior trauma increases vulnerability to PTSD [43].

Subsequent studies should aim to develop a more comprehensive sampling strategy that ensures robust representation across diverse sociocultural groups. Additionally, researchers should explore alternative resilience factors, such as self-efficacy and diverse support networks, that may moderate the association between exposure to prolonged traumatic events and PTSD. Identifying these factors can be achieved by mixed-methods studies with more active participation of adolescents. Future work should initiate repeated assessments shortly after exposure and at multiple follow-ups to map adolescent PTSD trajectories (resilient, recovery, chronic, delayed-onset) and their predictors, especially exposure severity, gender, media-related overwhelm, and resource loss, identified in war-affected cohorts [9, 10]. Such designs will clarify whether the high probable PTSD at one year reflects a chronically distressed subgroup under ongoing threat, delayed-onset cases, or elevations likely to remit as exposure abates. Such designs may also provide answers to the causal question regarding the association between health and PTSD among adolescents.

### Health policy implications

Our findings revealed that 41.9% of Israeli adolescents met the probable diagnostic threshold for PTSD one year after the October 7th attack – an alarmingly high prevalence underscoring the profound psychological consequences of prolonged conflict exposure. This striking figure is compounded by Israel’s chronic shortage of mental health resources, a vulnerability further exacerbated by the COVID-19 pandemic [44]. The stark contrast between these prevalence rates and the State Comptroller of Israel’s February 2025 report [45], which documented merely a 1% increase in mental health service utilization, highlights the magnitude of unmet need. This discrepancy may reflect the gap between earlier evaluations of mental health consequences [46], and the current realities faced by both adolescents and adults [45]. Taken together, the current crisis, coupled with our identification of critical PTSD risk factors, calls for urgent systemic reform and the comprehensive structural transformation of Israel’s mental health infrastructure.

In the short term, prevention efforts must prioritize widespread dissemination of PTSD symptom recognition and treatment accessibility information to both adolescents and their parents through integrated educational and community healthcare systems. Community-based primary care providers, including family doctors and pediatricians, represent a crucial but underutilized resource for early identification and intervention [46]. Comprehensive trauma-informed training programs should be implemented across all healthcare personnel to enhance recognition of trauma symptoms and delivery of evidence-based initial interventions [47, 48]. The integration of validated clinical assessment tools, including accessible self-report instruments, can systematically support this screening process [6].

Our findings linking economic hardship with PTSD highlight the urgent need for government interventions, particularly financial and housing support for families displaced, rendered homeless, or facing unemployment in the aftermath of the attack. Social workers should be mobilized to assist these families in addressing both the practical and emotional challenges associated with economic adversity.

Given our findings regarding the relationship between excessive media exposure and PTSD symptomatology, long-term policies should adopt evidence-based programs to promote coping strategies that have demonstrated efficacy across diverse age groups and trauma contexts [25, 49]. Empirical evidence suggests the effectiveness of targeted psychological support strategies for direct exposure [10, 50], and they should be adapted to the new era of indirect exposure with a severe impact. Furthermore, policy frameworks should support the development of clinical interventions that help adolescents engage in adaptive coping strategies, such as physical activity and peer engagement, while reducing reliance on maladaptive strategies, including self-blame and avoidance, to ensure effective and targeted mental health care [51]. These interventions must be developed with adolescents as active partners, ensuring that programs are responsive to their specific needs.

A particularly concerning finding from our research is the failure of resilience resources to mitigate the negative effects of identified risk factors, contrasting with patterns observed in previous traumatic events [52–54]. This deviation can be understood through the lens of resource depletion following prolonged traumatic exposure [51]. As posited by COR theory, resource loss correlates with stress and compromised mental health, with recovery dependent upon resource restoration [39, 40]. These findings, particularly regarding diminished social support networks, highlight the importance of long-term solutions, providing comprehensive mental support services based on family-centered and community-based interventions. Coordinated efforts between the Ministries of Health, Education, and Welfare are essential to support adolescents’ psychological recovery, promote family engagement, and sustain protective environments during protracted national crises.

## Conclusions

This study indicates several factors associated with post-traumatic stress symptoms among Israeli adolescents following the October 7th attacks, highlighting critical implications for health policy and service planning. Gender, poor health status, and prior adverse experiences emerged as salient background risk factors, pointing to the need for early identification of vulnerable youth within healthcare and school systems. The strong association between war-induced household economic decline and elevated PTSD symptoms underscores the importance of integrating mental health support with social welfare and economic assistance programs during prolonged emergencies. Additionally, the negative potential of overwhelming media exposure suggests the necessity of media literacy education and adult-mediated media engagement strategies as part of youth-focused mental health promotion. Although personal resilience and family support were protective, they did not buffer the impact of trauma exposure—indicating that resilience-building alone is insufficient without systemic, trauma-informed interventions.

Policy-makers should prioritize the development of scalable, culturally appropriate mental health services targeting adolescents, particularly in regions and populations disproportionately affected by conflict. Interventions should extend beyond direct trauma care to address indirect exposure and its compounding effects. Finally, urgent cross-sector collaboration among health, education, and social welfare systems is essential, with adolescents themselves positioned as active partners in shaping trauma-informed responses that are both effective and developmentally appropriate.

## Supplementary Information

Supplementary Material 1

## Data Availability

The datasets generated and analyzed during the current study are available from the corresponding author on reasonable request.
