# Supplementary material for: Post-traumatic stress among adolescents following the October 7th attack in Israel: implications for mental health policy and planning
Source: Isr J Health Policy Res. 2025 Dec 12;14:76. doi: 10.1186/s13584-025-00740-1 (PMC12699908; doi:10.1186/s13584-025-00740-1)
Supplement: Supplementary file 1 — Supplementary Material 1 [file 13584_2025_740_MOESM1_ESM.docx]

Appendix A
Interaction terms with resilience factors as moderators

| Independent variable (X) | Moderator (W) | *b* | *SE* | *p* |
| --- | --- | --- | --- | --- |
| War-related effects^1^ | Personal resilience | -0.69 | 0.73 | 0.34 |
| War-related effects^1^ | Family support | 0.35 | 0.44 | 0.42 |
| War-related effects^1^ | Friend support | -0.26 | 0.36 | 0.46 |
| Indirect exposure^2^ | Personal resilience | -0.14 | 0.20 | 0.47 |
| Indirect exposure^2^ | Family support | 0.79 | 1.11 | 0.48 |
| Indirect exposure^2^ | Friend support | 0.19 | 1.05 | 0.85 |

^1^ Sum of war-related effects, ranging from 0 to 5.

^2^ Equal to 1 if media exposure or overwhelming news consumption was reported.

Note. All coefficients are unstandardized. None of the interaction terms were statistically significant.
